# Supplementary material for: Are carbon emissions trading and green financial instruments synergistic? -Comprehensive quantitative research based on content analysis
Source: PLoS One. 2024 Mar 7;19(3):e0298601. doi: 10.1371/journal.pone.0298601 (PMC10919601; doi:10.1371/journal.pone.0298601)
Supplement: S1 Data — (ZIP) [file pone.0298601.s001.zip › Data/地级市Z_F工作报告.zip!/╣π╢1⁄2/│▒╓▌╩╨/└┤╫╘╣1⁄2╓┌║┼í╛┬φ┐╦╩2╛▌═°í┐_2011.docx]

各位代表：

现在，我代表市人民政府向大会作工作报告，请予审议，并请市政协委员和其他列席人员提出意见。

一、“十一五”时期的工作回顾

“十一五”时期，是我市经济社会发展最快、城市面貌变化最大、人民得到实惠最多的时期。过去五年，在省委、省政府和市委的坚强领导下，在市人大、市政协的监督支持下，市政府坚持以科学发展观为指导，紧紧依靠全市人民，抢抓机遇、改革创新，全力应对国际金融危机的冲击和影响，加快转变经济发展方式，着力拓展内外需求，切实保障和改善民生，积极构建和谐社会，有效保持了经济建设和社会事业平稳较快发展，较好完成了“十一五”规划和上次人代会确定的目标任务。

（一）经济综合实力进一步增强

经济平稳较快发展。“十一五”期间，全市经济发展情况良好，人民生活持续改善。去年，全市生产总值559.2亿元，比上年增长14.1%，“十一五”期间年均增长（下称年均增长）13.1%，比“十五”期间高出3.3个百分点。人均生产总值2.17万元，比上年增长13.6%，年均增长12.6%。三大产业结构从2005年的11.9：53.1：35调整优化为7.2：56.3:36.5。城镇居民人均可支配收入13669元，比上年增长10.3%，年均增长8.8%；农民人均纯收入6373元，比上年增长13%，年均增长7.6%。

财税金融情况良好。“十一五”期间，财税收入较快增长，金融形势保持稳定。去年，地方财政一般预算收入23.25亿元，比上年增长27.4%，年均增长21.9%。其中，非税收入3.51亿元，比上年增长99.9%。各项税收总额60.8亿元（含海关代征），比上年增长22.6%，年均增长17.7%。其中，国税国内收入35.91亿元、地税收入18.96亿元，分别比上年增长21%和18.5%，年均增长19.8%和17.4%。去年末，金融机构本外币存款余额653.3亿元、贷款余额219.4亿元，分别比上年末增长17.4%和13.9%，年均增长14.7%和9.9%。

县域经济发展壮大。“十一五”期间，区域发展协调性增强，县区经济加快发展。去年，潮安县、饶平县、湘桥区、枫溪区分别实现生产总值243.8亿元、133.8亿元、50.7亿元和70.8亿元，比上年增长15%、14%、13.5%和12.3%，年均增长14.7%、12.4%、10.8%和14.5%；地方财政一般预算收入分别完成6.6亿元、2.96亿元、2.05亿元和2.14亿元，比上年增长27.5%、25.4%、25%和17.1%，年均增长24.2%、24.4%、14.1%和14.3%。

（二）产业发展水平进一步提升

农业产业化程度提高。“十一五”期间，农业生产条件改善，现代农业园区、标准化示范区等建设成效明显，建成现代标准农田11.9万亩，全市粮食种植面积和总产量连续5年完成省下达计划。茶叶、潮州柑、花卉、蔬菜种植和深水网箱养殖等特色农业实现较快发展，规模化、机械化、产业化水平逐步提高。农业龙头企业和流通组织的作用进一步发挥，带动农业增产、农民增收。农业技术推广、标准化建设和农产品质量安全等工作加强，过去5年共获得无公害产品、绿色食品、有机食品认证176项，凤凰单丛茶地理标志产品保护获得国家批准实施，潮州柑地理标志证明商标成功注册。去年，全市农业总产值70亿元，比上年增长6%，年均增长3.9%。

工业发展能力不断增强。“十一五”期间，陶瓷、服装等八大优势特色产业加快发展，临港产业规模不断壮大，工业规模化、适度重型化趋势更加明显。去年，全市工业增加值284.3亿元，比上年增长18.2%，年均增长16.5%。其中，规模以上工业增加值183.7亿元，比上年增长20%，年均增长19.8%。规模以上企业1145家，年产值超亿元企业106家，分别比2005年增加417家和58家。欧华能源、长城集团、雅士利集团成功上市。临港产业建设不断推进，大唐潮州发电厂装机容量320万千瓦的1－4号机组全部建成投产，华丰燃气生产供应项目继续建设，大埕、所城、海山等风电场项目前期工作基本完成，一批大型企业即将落户，临港产业集聚效应逐步形成。全市轻重工业比例由2005年的63.2：36.8调整为58.9：41.1。

文化旅游产业长足发展。成立了市文物旅游局及相关机构，文化旅游管理体制得到理顺。古城文化旅游区保护建设成效明显，广济桥、牌坊街、许驸马府、己略黄公祠等一批重点文物全面修复开放，潮州文化旅游吸引力和竞争力全面提升。各具特色的自然生态景区得到开发，建成国家4A级景区3个，乡村旅游集群加快形成。木雕、潮绣、麦秆画等传统特色工艺生产逐步迈向产业化，旅游商品市场日趋活跃。潮州新八景评选活动反响巨大，旅游宣传推介和区域合作不断加强，我市成为省内最受欢迎的旅游目的地之一。去年，接待海内外游客357.7万人次，旅游收入54.2亿元，分别比上年增长16.3%和16.9%，年均增长16.9%和18.1%。

产业创新发展能力提升。科技创新平台建设加强，“十一五”期间国家企业技术中心建设实现零的突破，新增省级企业技术中心19家、省级工程研发中心14家、省级专业镇12个，累计达到20家、22家和17个，潮州陶瓷研究院、韩山师院陶瓷学院建成发挥作用，省市共建陶瓷先进制造业基地成效明显，省政府批准我市设立省级高新技术产业开发区。技术改造与创新步伐加快，“十一五”期间实施技改项目639项、完成投资80.3亿元，实施国家、省、市各类科技计划项目379项，列入省现代产业500强项目23个、省自主创新100强企业4家，认定高新技术企业38家、省级民科企业29家。质量标准战略较好实施，“十一五”期间设立陶瓷、食品、不锈钢等国家和省级质检机构7个，有60个单位主导或参与48项国家标准、26项行业标准和16项地方标准制修订。品牌建设成果丰硕，“十一五”期间创建国家级区域品牌18项、省级产业集群和特色产业基地10个，荣获中国驰名商标6件、中国名牌产品12项、省级名牌名标308项，累计拥有国家级区域品牌31个、省级区域品牌20个，省级以上名牌名标保有量348项。知识产权保护工作加强，“十一五”期间专利申请8249件、授权6561件，连续多年居全省各市前列。

产业园区建设成果丰硕。深圳（潮州）产业转移工业园被认定为省级示范园，获得省5亿元和深圳市2.5亿元资金扶持，连续两届招商推介会在深圳成功举办，已办理入园手续企业100家、投产52家。径南分园首期4500亩用地完成征收，园区基础建设加快进行。临港分园开发建设逐步推进，潮州港进港大道、亚太通用码头建设步伐加快，潮州港公用航道及西澳港区综合开发加紧进行，潮州港被国家列为对台直航港口之一。各县区产业园区规划建设工作积极开展。我市成为全省3个“产业转移工作和园区建设”双优秀的地级市之一。

（三）改革开放力度进一步加大

各项改革稳步推进。市级政府机构改革和第三轮行政审批制度改革全面完成，县（区）政府机构改革和第四轮行政审批制度改革加紧实施。财政管理体制不断完善，部门预算、集中支付、政府采购等改革全面推进。投融资体制改革逐步深化，组建了市金山投资开发公司、市潮发担保公司等投融资机构和信用担保机构。政府投资管理工作进一步加强，非经营性建设项目代建制得到落实。国有资产监管力度加大，国有企业改革继续深化。集体林权制度主体改革和基层农技推广体系改革全面完成，医疗卫生体制改革和教育、文化等重点领域改革取得新发展。

国内市场有效拓展。“广东潮州陶瓷暨特色产品（上海）展销会”等经贸活动成效明显，广东（潮州）赴北京商品大集被评为“新世纪十年全国优秀会展活动”，“潮货”知名度进一步打响。企业开拓市场力度加大，国内销售网络不断拓展，产品内销比重逐年提高。去年，全市规模以上工业销售产值707.2亿元，比上年增长23.3%。其中内销产值增长24.5%，所占比重从2005年的60.3%提高到76%。汽车下乡、家电下乡和以旧换新等鼓励消费政策得到落实，全社会消费品零售总额245.5亿元，比上年增长18.4%，年均增长19%。

对外贸易持续增长。外贸进出口经受住金融危机的冲击和影响，保持稳步发展的良好势头。去年，进出口总值38.2亿美元，比上年增长37.2%，年均增长15.4%。其中：出口总值23.4亿美元，比上年增长25.2%，年均增长9.9%；进口总值14.8亿美元，比上年增长61.5%，年均增长29.2%。陶瓷、服装、机电、鞋类、食品等重要产品出口稳定增长，出口市场更加多元化。全市出口超过1000万美元企业49家，比2005年增加16家。在发展对外贸易的同时，企业对外投资发展步伐加快，“十一五”期间全市共设立境外投资企业12家，累计13家。

招商引资成效明显。投资环境推介力度加大，“以商引商”和乡贤回归工程等较好实施，面向国内大型企业的招商活动积极开展，吸引外来投资工作取得一定成效。去年，新批外商投资项目36个，实际利用外资1.12亿美元，比上年增长11%。“十一五”期间，新增利用外资项目208个，实际利用外资4.55亿美元。中交集团投资开发的潮州港西澳港区、中国国电集团公司投资的新能源项目、广州港与亚太公司合作开发的亚太通用码头等一批重大项目计划总投资达到760亿元。

外事侨务有新发展。对外交流活动加强，各种互访明显增多，与法国巴黎第十三区缔结为友好城市，潮州在海外的影响力不断扩大。潮港、潮澳等交流合作不断加强，与海外社团的联系更加密切，广东省第二届粤东侨博会成功举办，荣誉市民授荣活动继续进行，“知侨、爱侨、护侨、重侨”氛围浓厚，侨捐资金管理进一步规范，旅外乡亲爱国爱乡热情迸发，“十一五”期间共接受旅外乡亲捐资3.43亿元。

（四）可持续发展能力进一步加强

投资力度有新增强。去年，全社会固定资产投资182.8亿元，比上年增长12.2%。投资结构不断优化，其中工业投资99.3亿元，比上年增长15.1%，占固定资产投资的54.3%；64个年度重点项目完成投资79亿元，占固定资产投资的43.2%。“十一五”期间，固定资产投资再创新高，累计完成投资712.2亿元，年均增长13.4%。其中，重点建设项目投资292.4亿元，占固定资产投资的41%。

基础建设扎实推进。“十一五”期间，基础设施建设累计完成投资294.8亿元。其中：交通建设完成投资65.4亿元，汕梅高速公路潮州段和省道335线、334线、233线、222线、231线潮州段等改造建设工程全面完成，厦深铁路潮州段及潮汕火车站进站公路、市客运中心枢纽站等建设正在推进，潮揭高速公路复工建设，镇通行政村公路硬底化建设提前一年完成省提出的目标要求；电网建设完成投资35亿元，超额完成“十一五”电力建设任务，新建11座110千伏以上输变电站和其他一批重点工程，电力保障能力全面提高；水利建设完成投资10.5亿元，韩江潮州供水枢纽建成蓄水，仙洲岛堤围整治和韩江南北堤达标加固等14宗水利防灾减灾工程，以及凤凰水库等6宗中型水库、102宗小型水库除险加固工程基本完成，沟尾溪、内洋南总干、河内湖三大涝区整治工程全部开工建设。

环保节能成效明显。国家环保模范城市创建活动积极开展，污染源普查工作全面完成，一批产能落后、污染严重企业被关闭。韩江、枫江、黄冈河流域污染综合整治稳步推进，大气污染治理力度加大，生态环境保持稳定，在省的环保责任制考核中连续多年获得优良等次。环境保护能力建设上新水平，市污水处理厂扩建工程完成，潮安、饶平县城污水处理厂建成投入使用，桥东污水处理厂建设加快，7个镇级人工湿地建成发挥作用，市城区污水处理率达到80%。市生活垃圾卫生填埋场一、二期工程建成，市区生活垃圾无害化处理率达到100%。林业生态工程建设力度加大，森林覆盖率达到60.8%，潮安县、饶平县先后通过林业生态县验收。节能节材、资源综合利用和陶瓷清洁生产等工作扎实推进，陶瓷窑炉节能降耗技术改造在全省推广应用。“十一五”期间，预计单位生产总值能耗下降16.09%，超额完成省下达的目标任务。

土地利用管理加强。新一轮土地利用总体规划修编完成，镇级国土资源所全部挂牌运作，耕地保护工作力度加大。节约集约用地水平提高，“三旧”改造积极开展，7个改造试点项目逐步推进。违法违规用地查处工作扎实有效，土地管理工作通过国家土地例行督察并受到好评。

人口计生工作水平提高。层级动态管理责任制落实，人口计生服务管理工作加强，计生利益导向机制逐步完善，低生育水平持续稳定。“十一五”期间，人口出生率、自然增长率均控制在省下达指标之内。湘桥区人口计生工作进入国家优质服务先进区行列，潮安县、饶平县进入省优质服务县行列。

（五）城乡发展面貌进一步改观

城市管理水平提高。城乡规划工作不断加强，《潮州市城市总体规划》等一批规划修编完成。城市管理权限下放，层级化责任体系初步形成，主要路段和重点地区整治成效明显，我市在国家城市综合整治定量考核中连续多年居全省前列。城市公交管理体制理顺，投入与管理力度加大，公交事业获得新发展。

城市功能不断完善。金山大桥建成通车，潮枫路、枫春路、开发区工业大道、春荣路、新洋路、中山路等道路改造全面完成，潮州大桥、韩江东西溪大桥奠基。桥东水厂、竹竿山水厂扩建工程顺利完成，城市供水范围不断扩大。西湖截污整治工程加紧实施。三利溪等排涝系统得到整治，市区积水问题基本解决。市文化艺术中心、市博物馆建成，市图书馆、市档案馆加紧建设。城市绿地和休闲设施增加，城市建成区绿地率38.3%、绿化覆盖率42.7%，绿化、美化、净化水平提高，荣获“国家园林城市”称号。韩江潮州河段环境整治与滨江景观建设，被建设部授予“中国人居环境范例奖”。

农村生活条件改善。新农村示范村建设成效明显，“万村绿”建设扎实推进，创建林业生态文明村156个。乡村公路建设取得新发展，行政村通班车覆盖率达到95%。15.28万人的农村饮水特别困难问题全部解决，饶平沿海六镇饮水解困工程完成，农村饮水安全工程建成45宗、受益61万人。新型农村合作医疗继续发展，参合率提高到99.89%。农村养老保险试点工作启动，参保人数达到6.53万人。扶贫开发“规划到户、责任到人”成效明显，有3872户、16608人实现稳定脱贫，脱贫率达到51.7%。

（六）和谐社会建设进一步推进

社会保障工作加强。去年，城镇登记失业率2.33%，低于全省平均水平。“十一五”期间，累计增加就业岗位18.9万个，全社会非农就业比重超过76%。农村劳动力培训和转移步伐加快，累计培训7.86万人、转移就业21.7万人。社会保障体系不断完善，城镇养老、医疗、失业、工伤参保人数达到39.1万、39.8万、31.4万、30.3万，分别比2005年增长49%、874%、33.7%和94.5%。养老保险待遇大幅度提高，企业退休人员人均每月养老金950元，比2005年增加581元。4.5万名困难国有集体企业退休人员得到资助纳入医保范围。

文教体卫全面发展。城市公共文化设施不断完善，乡镇文化站达标率大幅度提高，农家书屋建设超额完成省下达任务，广播电视村村通工程扎实推进。文物和非物质文化遗产普查工作完成，全市不可移动文物景点1390处，列为国家级、省级、市级“非遗”保护项目分别为11项、24项、28项，“潮州土楼”成为国家第三次文物普查“十大新发现”之一。一批工艺美术人才脱颖而出，拥有省级以上工艺美术大师58名，居全省各市首位。城乡免费义务教育政策全面落实，高中阶段毛入学率达到84.8%，市金山中学等5所学校被评为国家级示范性高中；中小学代课教师问题全面解决，教师福利待遇逐步提高，基础教育在全省责任制考核中获得优秀等次；职业技术教育加快发展，市卫校、市职校、市技工学校新校区建成投用，市卫校、市技工学校被评为国家级重点中专学校。群众性体育设施不断完善，形式多样的体育活动蓬勃开展，亚运火炬传递圆满成功。公共卫生服务体系逐步健全，基层卫生服务加快发展，疾病防控能力有新增强。

社会福利水平提高。最低生活保障覆盖面扩大，去年低保人数70795人，比2005年增加22171人。住房解危解困工作扎实推进，改造农村贫困户危房14212户，落实城镇廉租住房实物配租857户、租赁补贴939户。社会慈善活动广泛开展，“春风暖人心”一系列爱心工程积极实施，扶危济困渐成风尚。对口援建汶川耿达乡工作成效明显，“广东潮州新村”等项目建设全面完成。

社会大局和谐稳定。公安“三基”工程建设全面达标，视频监控网点不断健全，治安防控能力显著提高，违法犯罪活动受到有效打击。市、县区主要领导信访接待日成效明显，各级信访大厅达标建设和综治信访维稳中心建设全面完成，信访“三无”村镇创建活动扎实开展，群体性事件得到妥善处置，综治工作连续多年在省考核中获得优秀等次。市场秩序不断规范，产品质量和食品药品安全水平提升。安全生产形势持续稳定，应急管理工作逐步加强。

其他工作全面发展。文明创建活动广泛开展，连续5次荣获“广东省文明城市”称号。“五五”普法工作全面完成，双拥共建、人民武装、人事编制、粮食、物价、审计、统计、打私、人防、气象、防震、地方志、档案、老龄、民族宗教、人文社科、妇女儿童、残疾人和红十字等工作不断取得新进步。

在经济社会取得新发展的同时，政府自身建设继续加强，法制建设加快推进，行政管理和服务水平不断提高，“一站式”行政服务逐步完善。接受市人大、市政协监督的自觉性增强，“十一五”期间共办理市人大代表建议253件、市政协提案432件，代表和委员满意和基本满意率较高。与各民主党派、工会、共青团、妇联的联系加强，科学民主决策机制不断完善。政务公开扎实推进，电子监察系统建立健全，行政审批网上办理率去年跃升为全省第3名，政风行风热线节目效果良好。政府廉政建设责任制全面落实，一批违法违纪案件受到严肃查处。

各位代表，过去五年，我市能够有效地应对国际金融危机冲击，较好地保持经济社会平稳较快发展，主要得益于我们紧紧把握好以下要点：一是坚持解放思想、改革创新。始终紧紧把握好发展这个第一要务，坚持解放思想、与时俱进，积极推进改革发展，有效地应对了各种挑战。二是坚持调整结构、转型升级。在应对危机保增长的同时，遵循产业发展和市场经济规律，大力加强产业建设，积极推动创新发展，努力在调整中抓转型促升级，在发展中打基础增后劲。三是坚持统筹兼顾、以人为本。把推动经济发展与保障改善民生有机结合起来，把更多的财力物力用在经济社会协调发展和人民生活水平提高上，促进了经济良性循环与社会和谐稳定。四是坚持依法行政、转变作风。以深入学习实践科学发展观活动和“落实推进年”活动为动力，切实转变作风，强化责任落实，提高工作效能，有效地推进各项工作发展。

各位代表，“十一五”期间取得的成绩来之不易。这些成绩的取得，是省委、省政府和市委正确领导的结果，是历届市委、市政府团结带领广大人民群众辛勤努力、奋发图强的结果，是市人大、市政协有效监督和大力支持的结果，也是驻潮部队、武警官兵、上级驻潮各单位，以及港澳台同胞、海外侨胞和社会各界鼎力相助的结果。在此，我代表市人民政府表示衷心的感谢并致以崇高的敬意!

在看到成绩的同时，我们也清醒地认识到，我市经济社会发展中还存在一些不容忽视的困难和问题。一是经济总量小，改革创新发展的能力不强，跨越式发展和科学发展的压力与挑战巨大。二是经济结构性矛盾突出，优势特色产业不强，新兴产业发育不足，第三产业所占比重较低，金融机构存贷差额加大。三是土地、资源、环境制约日益明显，节能减排任务依然艰巨。四是开拓创新精神不够，行政效能和服务水平不高等问题比较突出。这些问题，有待于我们在今后的工作中加以解决。

二、“十二五”时期发展目标和重点工作

“十二五”时期，是我市加快转变经济发展方式、实现“五年大变化、十年大发展”的关键时期，也是进一步夯实基础、增强后劲、提升优势、跨越发展的重要阶段。实现我市经济社会加快发展、科学发展、和谐发展，既面临许多机遇，也存在不少挑战，但总体上机遇大于挑战。我们一定要认清新形势、适应新变化，立足新起点、完善新思路，抓住新机遇、推动新发展，全面实现“十二五”规划发展目标。

“十二五”时期，全市经济社会发展的指导思想是：坚持以邓小平理论和“三个代表”重要思想为指导，深入贯彻落实科学发展观，始终把握科学发展这个主题和加快转变经济发展方式这条主线，围绕“加快转型升级、建设幸福潮州”的总体要求和实现“五年大变化、十年大发展”的奋斗目标，大力发展特色经济、文化经济、港口经济，不断保障和改善民生，努力保持经济平稳较快发展和社会和谐稳定，着力打造国内外有重要影响力的历史文化名城、国内重要的特色产业基地、广东重要的临港产业基地，全力建设宜业宜游宜居的现代化滨江城市。

“十二五”时期，全市经济社会发展的主要目标是：生产总值年均增长15%，人均生产总值年均增长14.5%，地方财政一般预算收入年均增长18%，固定资产投资年均增长20%，外贸出口年均增长10%，社会消费品零售总额年均增长20%，城市居民人均可支配收入年均增长10%，农村居民人均纯收入年均增长12%，年人口自然增长率控制在6.6‰以内，年城镇登记失业率控制在3%以内，2015年单位生产总值能源消耗比2010年降低16%、城镇化水平达到67%。到“十二五”期末，全市经济社会发展达到全省平均水平，实现“十年大发展”基本要求。

根据上述指导思想和主要目标，要统筹兼顾、突出重点抓好以下工作：

（一）调整优化产业结构，着力构建现代产业体系。坚持做优第一产业、做强第二产业、做大第三产业，以现代农业为基础，以特色产业为依托，以临港产业为重点，以战略性新兴产业为引领，以现代服务业为配套，全力加强产业建设，努力构建布局合理、结构优化、技术先进、集约节约的现代产业体系。

（二）增强自主创新能力，着力提升产业整体素质。坚持把科技进步和创新作为加快转变经济发展方式的核心推动力，强化企业创新主体地位，集聚创新资源，完善创新体系，营造创新环境，增强创新能力，加强知识产权保护，实施质量强市战略，大力推进品牌和技术标准建设，全面提高信息化水平，促进自主创新能力和产业竞争能力“双提升”。

（三）实施大项目带动战略，着力促进经济跨越式发展。坚持积极利用国内外资源，加强投资环境宣传推介，抓好项目包装策划，创新招商引资办法，大力引进和实施一批关系全局的重大项目。积极拓宽融资渠道，加大财政统筹和投入力度，全力推进基础设施建设，为经济社会加快发展创造良好的环境和条件。

（四）培育扩大消费需求，着力拓展内需增长空间。坚持扩大内需战略，充分挖掘内需市场潜力，发挥投资的拉动作用，加快形成消费、投资、出口协调发展的新局面。整顿和规范市场秩序，加强食品安全和产品质量监管，着力营造诚实守信的社会环境和市场环境。

（五）促进文化与经济融合，着力提升潮州文化软实力。坚持以文化旅游为龙头，以修复一批文物景点、振兴一批传统工艺、建设一批文化设施、创作一批文化精品、壮大一批文化产业“五个一批工程”为重点，加快推进文化与经济融合，全力把我市打造成为全省优秀文化传承先行地、提升区域文化软实力主导区、文化与经济融合示范市。

（六）加快城市化进程，着力建设“三宜”滨江城市。坚持以规划为引领，以基础建设为依托，加快拓展城市发展空间，统筹协调城乡发展，优化农村发展环境，完善城乡一体化布局，提高城市化发展水平，建设宜业宜游宜居的现代化滨江城市。

（七）加强资源环境保护，着力增强可持续发展能力。坚持把建设生态文明作为转变经济发展方式的重要着力点，树立绿色、低碳发展理念，以节能减排为重点，健全激励和约束机制，推进资源节约集约利用，加强环境保护和生态建设，努力改善环境质量，增强可持续发展能力。

（八）继续深化改革开放，着力建立开放型经济体系。坚持以推进重点领域和关键环节改革为重点，破解经济社会发展的体制障碍，增强发展内生动力。优化对外贸易结构，提高招商引资水平，促进对外交流与合作，努力提高开放型经济水平。

（九）不断改善民生质量，着力保持社会和谐稳定。坚持民事为大、民生为重、民安为乐、民心为上，优先发展教育事业，千方百计扩大就业，完善社会保障体系，提高全民健康水平，促进各项社会事业全面发展，努力提高社会福利和公共服务水平，切实保障和改善民生，全力建设幸福潮州。

三、2011年的工作意见

今年是“十二五”的开局之年，也是我市实现“五年大变化”的决胜之年。我们既积累了加快发展的有利条件，也面临着制约发展的不利因素。从大环境看，后金融危机的影响并没有完全结束，国际贸易保护主义更加明显，国内宏观调控政策趋紧、通胀压力较大，我市产业结构矛盾突出、要素约束增大，这对持续较快发展产生很大压力。但是，省委、省政府加快粤东地区振兴崛起战略决策的实施，我市拥有的资源优势、比较优势和后发优势等，有利于我们加快转变经济发展方式，深入推进科学发展。我们要准确把握国内外形势，充分利用各种有利条件，积极克服各种不利因素，进一步提高工作的针对性和实效性，努力促进经济社会加快发展。

根据“十二五”发展规划，并综合考虑各种因素，今年全市经济社会发展预期目标是：生产总值增长14%，单位生产总值能耗下降3.43%，地方财政一般预算收入增长15.5%（新口径），固定资产投资增长20%，外贸出口增长15%，社会消费品零售总额增长18%，居民消费价格指数涨幅控制在3.5%以内，人口自然增长率控制在6.58‰以内，城镇登记失业率控制在3%以下，市区居民人均可支配收入增长10%，农村居民人均纯收入增长12%。

为实现上述目标，我们要深入贯彻落实科学发展观，按照中央经济工作会议、省委十届八次全会和市委十二届十次全会的部署要求，以科学发展为主题，以加快转变经济发展方式为主线，以保持经济平稳较快增长为主攻方向，以转变作风、强化落实为重要抓手，加快调整优化产业结构，大力推进创新发展，努力扩大对外开放，着力保障和改善民生，全力建设幸福潮州。重点抓好以下八个方面工作：

（一）狠抓“三农”工作，努力提高农村发展水平

大力发展现代农业。推进现代标准农田建设，加快中低产田改造，千方百计稳定粮食生产，确保全年粮食种植面积66.5万亩、总产26.4万吨。下大力气抓好“菜篮子”产品生产，提高自我供应保障能力。扩大茶叶、花卉、水果、蔬菜等种植规模，做大做优“潮州柑”、“凤凰单丛茶”品牌，支持铁皮石斛国家中药现代化科技产业基地发展，推动特色农业向现代农业转变。完善海洋与渔业基础设施，加快标准化渔港建设，大力发展深水抗风浪网箱养殖。继续培育壮大农业龙头企业，大力发展农民专业合作社和流通组织，加强农业示范基地、现代农业园区和健康养殖示范区建设，提升农产品质量档次，畅通农产品流通渠道，提高农业生产经营效益。

努力促进农民增收。落实各项强农惠农政策，加大“三农”投入，促进农业增产、农民增收。支持农村土地承包经营权依法流转，鼓励农民发展适度规模经营，积极挖掘农业内部增收潜力。加强农村劳动力技能培训，提高农民职业技能和创收能力，推动农民通过转移就业增加收入。着力改善农村创业环境，引导农民开展生态、休闲、观光等农业经营活动，发展农产品加工、保鲜、运销、服务等二三产业，多渠道增加农民收入。

加快改善农村环境。全力加强水利防灾减灾工程和农田水利设施建设，加快病险水库、水闸的除险加固，抓好小流域综合整治和重点灌区改造，推进河内湖和内洋南总干排涝整治工程，完成沟尾溪工程建设。重视农村沟渠湖塘清淤疏浚，营造河畅、水清、岸绿、景美的水环境。抓好畜禽养殖污染防治，推进农村垃圾集中处理和污水处理。加快社会主义新农村示范点建设，开展环境优美乡镇、生态示范村创建活动，建设农民美好家园。

（二）促进产业转型升级，努力提升产业整体素质

推进产业创新发展。积极构建公共技术研发平台，加强特色产业基地建设，促进专业镇转型升级。着力培育企业技术创新机构，加强工程研发中心、企业技术中心等研发载体建设，力争新建一批省级以上研发机构。加快传统产业改造步伐，支持企业加强技术攻关和技术改造，努力提升优势特色产业发展水平和自动化、半自动化生产水平。扶持发展一批创新型企业，鼓励开发和引进新材料、新技术、新设备。推进产学研合作，扩大产学研合作效应。加强知识产权培育和保护，促进知识产权工作上新台阶。

实施质量强市战略。构建符合创新发展和民生取向的质量工作机制，促进产品质量、工程质量、服务质量、环境质量水平不断提升。支持企业参与各类标准的制修订，引导企业采用国际标准或国外先进标准组织生产。加快省鞋类质检站、水族器材质检站、凤凰茶叶检测实验室等质检机构建设，争取食品软包装材料国家中心获得批准筹建。完善品牌培育机制，创建更多的名牌名标，争取“庵埠食品”集体商标注册成功。重视品牌经营保护，以品牌带动产业发展。

壮大产业经济规模。落实鼓励企业上市的政策措施，加大对上市后备资源的培育力度，促进三环、凯普、正龙等企业尽快上市。引导企业创新经营理念和发展模式，抓好规模以下企业转化工作，促进更多企业发展壮大。发挥园区的产业集聚优势，推动优势特色产业规模化、集群化、高端化发展。加强信用担保机构建设，积极帮助企业解决贷款难、担保难问题，推进企业做大做强。

培育高新技术产业。加紧做好高新区的规划，完善管理体制，加强基础建设，优化发展环境，努力把高新区建设成为科技创新示范、科技成果转化、高新技术产业化基地。加快制订产业培育规划，突出重点扶持一批骨干企业和投资项目，培育发展一批优势企业和高新技术企业，形成经济发展的先导产业和支柱产业，力争新增高新技术企业10家。以三环、凯普和响石等企业为龙头，带动电子信息、生物制药、数字视听、机械制造、新光源照明等高新技术产业和先进制造业发展。

（三）加快临港产业发展，努力发展壮大港口经济

加强港区基础建设。尽快完成潮州港及潮州港经济区总体规划，以及重化工业园、产业转移园控制性详细规划，加强各类规划的衔接完善，切实抓好港口岸线资源和土地资源的保护管理。加快港口港区基础设施建设，力争进港公路主体工程建成、亚太通用码头一期工程基本完成，推进潮州港公用航道与西澳港区综合开发建设，完成小红山5000吨级多功能货运码头续建。抓紧做好潮州港供水工程前期工作，争取疏港铁路纳入国家规划。

推进临港产业发展。加大协调力度，保障大唐发电厂1－4号机组安全运行，着手开展5、6号机组建设前期工作。大力推进国电、华能风能电场等项目建设，抓好和洋船舶配套产品基地等项目筹建，推进对台直航工作，促进临港产业发展壮大。抓住国家加快发展战略性新兴产业的有利时机，争取在新技术、新材料、新能源和生物环保等新兴产业发展上有所突破，积极发展能源石化、装备制造、船舶修造等产业，推动全市经济结构战略性调整。

加大力度招商引资。大力宣传推介潮州港的资源优势和发展潜力，提高潮州港的投资吸引力。加强项目策划包装，创新招商方式，主动联系国内外大型企业，选择引进符合国家产业政策、带动能力强的重大项目。加强跟踪服务和协调配合，努力提高投资项目的履约率。借助西澳港区的综合开发，积极推进招商引资工作。抓紧做好临港产业转移园首期2700多亩土地的征收储备，为招商引资和项目落地创造条件。

（四）发展文化旅游产业，努力促进文化与经济融合

建设古城文化旅游区。加快完成古城区控制性详细规划，推进古城文化旅游区的保护、利用和管理。以创建5A级景区为目标，进一步完善广济桥、牌坊街配套设施，将牌坊街申报为中国历史文化名街，启动笔架山宋窑遗址公园的规划建设和韩祠橡木园修建工程，加快海外潮人博物馆建设，配备古城区旅游电瓶专线车，依托牌坊街开设工艺品集散市场、特色美食旅游餐饮区，不断丰富古城区文化旅游内涵。

促进旅游业全面发展。推进环古城旅游资源开发，将绿岛山庄、地质冰川公园等景区景点整合为大型休闲生态旅游景区，加快凤凰山、紫莲山庄、绿太阳山庄、凤翔峡、幽谷逸林等生态景点转型升级。精心设计观光、休闲、娱乐和购物一体化的旅游线路，推行并延伸古城旅游“优惠套票”，提高旅游产业的综合效益。加强旅游整体形象策划，创新宣传推介方式，举办“潮州行”系列旅游推介活动，强化与外地旅行社的联系合作，推动外地旅行社送客入潮。加大高星级酒店的招商引资力度，力促国际大酒店、潮安迎宾馆等尽快建成投入使用，鼓励发展民居客栈，提升旅游接待能力。

加快发展文化产业。大力整合民间传统文化资源，鼓励运用现代技术和理念改造提升传统的创作、生产、传播方式，促进潮绣、木雕、麦秆画等传统工艺商品化、产业化、规模化发展，做大做强传统文化产业。加快陶瓷文化创意产业园等建设，促进文化创意、工业设计、数字传媒、印刷发行等新兴文化产业发展，形成多门类文化产业竞相发展的局面。

（五）坚持投资消费拉动，努力保持经济较快增长

全力加快重点项目建设。创新项目建设思路，拓宽融资渠道，着力解决项目征地拆迁难、资金筹措难等问题，确保年度投资107.5亿元的61个重点项目顺利实施。抓住省掀起粤东西北交通建设大会战的有利时机，推进厦深铁路、潮揭高速公路、潮惠高速公路潮州段、潮汕火车站进站公路和韩江东西溪大桥建设，加快县道砲浮线等道路改造，抓好220千伏线路工程和110千伏输变电工程等电力重点项目建设。加紧做好“十二五”前期投资项目的筹划工作，争取一批项目提前开工建设。重视政府投资导向作用，研究出台鼓励民间投资的措施，带动民间投资发展，全力扩大投资规模。

努力开拓和培育国内市场。筹划在国内重点城市设立潮州特色产品专馆，举办多形式特色产品展销会，组织企业参加各类经贸活动，努力提高“潮货”知名度和影响力。积极引导企业调整产品结构，生产适销对路的产品投放国内市场。支持生产企业与大型营销企业合作，通过代理制或设立直销店、专卖柜等形式，建立新的营销网络和销售渠道，全力提高“潮货”的市场占有率。

加强市场监管和物价监测。整顿规范市场秩序，严厉打击制售假冒伪劣产品违法行为，强化对定点医疗机构和定点零售药店的监管，提高产品质量和食品药品安全水平。经常性开展市场价格检查，及时发布物价信息，正确引导社会预期，整治串通涨价、哄抬物价等不法行为，努力控制物价上涨幅度。

积极培育发展现代服务业。遵循“产业推动市场，市场带动产业”的发展规律，加快推进潮州国际陶瓷交易中心、市陶瓷原料配送中心、潮汕火车站区物流中心、粤东水产品物流中心等项目建设，着手抓好纺织服装、食品、不锈钢、鞋类等产品和原材料专业市场建设，为特色产业发展创造良好条件。适应消费结构升级趋势，大力发展连锁经营等新型流通业态，引导商贸连锁经营向县城、乡镇延伸，做大做强商贸流通业。积极发展经济监证、会计服务、信息咨询等中介服务业，以及休闲娱乐、卫生保健等新兴服务业，引导产业联动发展。

全面提升开放型经济水平。针对国际贸易保护主义常态化、人民币升值和招工难等问题，引导企业增强危机意识，用好用活各项扶持政策，重视技术和产品创新，努力培育出口品牌，优化出口市场结构和产品结构，不断增强应对能力。支持企业加快“走出去”步伐，设立更多境外投资公司，充分利用境外资源和先进技术发展生产、扩大经营。创新引资方式，完善发展载体，健全项目储备，强化包装推介，力争引进一批优质大项目。积极参加省组织的面向中央企业、大型民营企业和世界500强的三场大型招商活动，推进与国内外大型企业合作，努力提高开放型经济发展水平。

（六）加快城乡统筹发展，努力推进城市化进程

坚持科学规划引领。完成《潮州市城市总体规划（修编）》报批并认真抓好实施。开展韩江东岸溪南片、溪北片、意东综合片、潮揭高速南片等重点区域控制性详细规划的编制。推动潮州大桥建设，畅通韩江东西片区的连接，规划建设配套路网，拓展城市发展空间；重视城市东片区大型设施布点，开发建设意东综合片区，带动和促进城市跨江发展。加大监察执法力度，坚决查处违法违规建设行为。

加强城市建设管理。按照“把握节点、突出重点、打造亮点”的原则，全力打造良好城市形象。突出重点加快推进银槐北路、外环路，以及绿榕南路等一批道路建设，辟建2个新的停车场，改善城市交通环境。加强道路绿化美化，改造提升植物园、西湖公园，提高国家园林城市建设水平。完善公共服务设施，完成市图书馆、档案馆建设。加大城市保洁力度，建设一批垃圾压缩站。

加快产业园区建设。落实深圳潮州合作共建机制，全力推进深圳（潮州）产业转移园建设发展，重点加快径南分园建设，合理规划功能分区，争取年底前完成3000亩土地开发。加快推进市开发区二期B区和各县区产业园的规划建设，引导工业向园区聚集。配套完善园区的生活服务设施，努力把产业园区建设成为城市工业新区。

推进节约集约用地。认真实施新一轮土地利用总体规划，严格土地投资强度、建筑容积率等指标，规范已批建设用地管理，加大闲置土地处置力度，遏制盲目圈地和低水平建设，努力盘活土地资源。加快“三旧”改造步伐，力争市区“三旧”改造面积1270亩以上。落实国土管理共同责任制，坚决查处违法违规用地占地和违法开采矿产资源等行为。

抓好节能环保工作。加快节能新技术、新工艺、新产品的推广应用，加强建筑业、交通运输业和公共机构等节能工作。大力发展循环经济，积极发展资源节约和综合利用项目。推进国家环保模范城市创建工作，严格环境监管和准入制度，整治环境污染突出问题，加强主要污染物排放总量控制，重视海洋资源和环境保护。搞好韩江、黄冈河等饮用水源保护工作，确保饮水安全。实施引韩济枫工程，加快桥东污水处理厂等项目建设，搞好雨污分流改造，提高污水处理水平。推行种植业、林果业与畜牧业、水产养殖业有机结合的生态种养方式，减少生态污染。实施沿海防护林及红树林、韩江流域水源涵养林等林业生态工程，完成林业生态市建设任务。

（七）重视民生保障工作，努力促进社会和谐发展

扎实推进各项改革。加强税收征管服务，公平税负、培植税源。调整优化支出结构，严格控制一般性支出，加大民生投入力度，着力提高群众幸福指数。深化部门预算管理、事业单位人事分配制度和文化体制等重点改革。推进投融资体制改革，创新投融资办法和途径，拓宽资金筹措渠道。加强金融服务与监管，增强金融对经济的支撑力度，搞好新型农村金融机构组建和农信联社股权改造。深化集体林权制度改革，巩固扩大林改成果。

积极为民办实事。把纾民生、解民困工作抓实抓好，集中力量办好十件好事实事。一是千方百计促进就业。加强城乡公共就业服务，强化职业技能培训，重视城乡统筹就业，拓宽就业渠道，新增城镇就业3万人，下岗失业人员再就业8000人，城镇登记失业率控制在3%以内，组织农村劳动力培训2万人、转移就业5万人。二是推动教育公平均衡发展。将农村义务教育阶段学生生均公用经费标准提高200元，中职学校、技工学校免学费政策扩大至城市家庭经济困难学生；优化中小学校布局，逐步完善新城区、县城区及中心镇学校配套；推进中小学校规范化建设，确保覆盖率达到50%；大力发展学前教育，力争学前三年毛入园率达到90%；加快发展职业教育，全面完成普高任务，确保高中阶段毛入学率达到86%以上。三是促进医疗卫生服务均等化。认真落实医改各项政策，全力推进医疗卫生信息化建设，逐步实现全市居民健康档案电子化，动态建档率达到60%；政府办基层医疗卫生机构100%实施基本药物制度；继续推进宫颈癌免费普查工作，争取普查10万人以上；落实新型合作医疗和城镇居民医保补助标准，确保新农合参合率提高到99.9%、城镇基本医疗保险覆盖率达到65%。四是抓好住房解困保障工作。增加城镇保障性住房952套，其中实物配租56套、租赁补贴420户、经济适用房430套、公租房46套；改造农村低收入家庭危房3000户；加强房地产市场管理，增加中低收入居民住房供给。五是加大扶贫开发力度。实现3962户贫困户稳定脱贫，全面完成扶贫开发“双到”任务；设立扶贫救济专项资金，对1028户贫困户实施扶贫救济，每户每年救助1000元。六是搞好农村饮水安全工程。全面完成65.57万人的农村饮水安全工程建设任务；抓紧开展10.7万人的饮水解困工程前期工作，争取尽快实施并早日完成。七是改善农村出行条件。新建县乡公路50公里、通村公路250公里，提高农村通畅度；大力发展农村客运，新建改建乡镇客运站17个、候车亭194个，符合通行客运班车安全条件的行政村100%通行客运班车。八是大力发展城市公共交通。完成市客运中心枢纽站建设，大力整治违法违规客货运输站点；新增或延长公交线路5条，新增公交车辆50部，提高公交线路覆盖面。九是加强社会救助援助工作。投入1234万元向全市困难群众发放临时性价格补贴，继续实施“春风暖人心”爱心工程，开工建设市老年康复护理中心，启动饶平县、湘桥区残疾人综合服务中心建设，免除0-6周岁贫困残疾儿童的康复训练经费和辅助器具所需经费。十是推进城乡文化设施建设。创建国家级潮州文化生态保护区，完成234个农家书屋建设任务，确保全市文化站建设全面达标，实现20户以下已通电自然村广播电视村村通。

统筹发展社会事业。建立城乡居民最低生活保障标准动态调整机制，实现应保尽保；抓好城镇职工社保扩面征缴，大力推进新型农村养老保险工作；提高企业退休人员基本养老金，全面解决市属国有困难企业欠缴养老保险费问题；探索城镇职工养老保险、城乡居民养老保险衔接办法，畅通养老保险异地转移通道。规范基层人口计生服务管理，加强县级计生服务站和8个镇级中心服务所建设，突出抓好农村和流动人口计生工作，完善计生利益导向机制，稳定低生育水平，力争人口计生工作保持在全省先进水平。搞好村和社区两委换届选举工作。继续做好人才、民族宗教、新闻出版、对台、人防、气象、防震、统计、档案、地方志、妇女儿童、老龄和残疾人等工作，促进各项事业全面发展。

维护社会和谐稳定。深入开展群众性文明创建活动，启动“六五”普法宣传。加强城乡和谐社区建设，推进社区服务工作。创新信访机制，拓宽信访渠道，引导群众依法表达利益诉求。落实企业安全生产主体责任、政府部门安全监管责任，抓好重点领域和重点行业安全专项整治。加强应急管理工作，提高保障公共安全和处置突发事件的能力。重视国防教育、国防动员、民兵预备役和人民防空工作。加强矛盾纠纷排查化解，完善社会治安管理机制，维护社会稳定。

（八）加强政府自身建设，努力增强政府执政能力

重视创新发展。坚持解放思想、与时俱进，以创新的精神、务实的作风、高效的举措，不断提高谋划发展、统筹发展和推动发展的能力，全力促进政府工作上新水平。

推进依法行政。健全行政决策程序，规范行政执法行为，强化过错责任追究，提高依法依规办事水平。深入推进政务公开，增强政府工作透明度和公众参与度。自觉接受市人大及其常委会和市政协的监督，认真办理人大代表和政协委员的议案、建议和提案。充分发挥各民主党派参政议政作用和工青妇等人民团体的桥梁纽带作用，虚心听取各方面意见。

加快职能转变。推进第四轮行政审批制度改革，简化审批环节，提高行政效率，集中精力保民生、促发展。完成县区政府机构改革，加强各级行政服务中心建设，完善网上审批和为民服务全程代理制，推进服务型政府建设。建立健全部门协调配合机制，努力推进各项工作落实。

加强廉政建设。全面落实党风廉政建设责任制，不断完善惩治和预防腐败体系。加强行政监察和审计监督，办好政风行风热线，坚决纠正行业不正之风。积极推进公务员队伍建设，全面提升公务员综合素质，树立廉洁高效的公务员形象。

各位代表，走过了“十一五”不平凡的历程，踏上了充满希望和挑战的新征程。让我们在省委省政府和市委的坚强领导下，振奋精神、锐意进取，团结拼搏、扎实工作，为实现“十二五”发展开好局起好步、全面完成今年各项目标任务、建设幸福潮州而努力奋斗！
